# Facial Contrast Is a Cross-Cultural Cue for Perceiving Age

**DOI:** 10.3389/fpsyg.2017.01208

**Published:** 2017-07-25

**Authors:** Aurélie Porcheron, Emmanuelle Mauger, Frédérique Soppelsa, Yuli Liu, Liezhong Ge, Olivier Pascalis, Richard Russell, Frédérique Morizot

**Affiliations:** ^1^Chanel PB Pantin, France; ^2^Laboratoire de Psychologie et NeuroCognition, Université Grenoble-Alpes Grenoble, France; ^3^Lincoln Group Boulogne-Billancourt, France; ^4^Department of Psychology, Zhejiang Sci-Tech University Hangzhou, China; ^5^Department of Psychology, Gettysburg College Gettysburg, PA, United States

**Keywords:** facial contrast, age, face perception, races, cross-cultural

## Abstract

Age is a fundamental social dimension and a youthful appearance is of importance for many individuals, perhaps because it is a relevant predictor of aspects of health, facial attractiveness and general well-being. We recently showed that facial contrast—the color and luminance difference between facial features and the surrounding skin—is age-related and a cue to age perception of Caucasian women. Specifically, aspects of facial contrast decrease with age in Caucasian women, and Caucasian female faces with higher contrast look younger (Porcheron et al., 2013). Here we investigated faces of other ethnic groups and raters of other cultures to see whether facial contrast is a cross-cultural youth-related attribute. Using large sets of full face color photographs of Chinese, Latin American and black South African women aged 20–80, we measured the luminance and color contrast between the facial features (the eyes, the lips, and the brows) and the surrounding skin. Most aspects of facial contrast that were previously found to decrease with age in Caucasian women were also found to decrease with age in the other ethnic groups. Though the overall pattern of changes with age was common to all women, there were also some differences between the groups. In a separate study, individual faces of the 4 ethnic groups were perceived younger by French and Chinese participants when the aspects of facial contrast that vary with age in the majority of faces were artificially increased, but older when they were artificially decreased. Altogether these findings indicate that facial contrast is a cross-cultural cue to youthfulness. Because cosmetics were shown to enhance facial contrast, this work provides some support for the notion that a universal function of cosmetics is to make female faces look younger.

## Introduction

Age is a fundamental social dimension that drives social behaviors in professional and personal contexts. It is not only *actual* or *chronological* age that predicts a person's treatment by others; the *apparent* age is a major motivating factor for many individuals, though there are evident marked cultural differences in the amount of emphasis placed on the apparent age of social partners (Anzures et al., 2010). A youthful appearance is of importance for many individuals, perhaps because it is a relevant predictor of aspects of health and facial attractiveness (Christensen et al., 2004; Rhodes, 2009).

Facial cues that drive youthful appearance have been shown to share similarities across different races, notably because faces age in similar ways across different races (Tschachler and Morizot, 2006; Hillebrand et al., 2007; Farage et al., 2010). Skin texture (wrinkles) and skin color homogeneity (hyper-pigmentation) are good predictors of perceived age in Caucasian (Nkengne et al., 2008; Gunn et al., 2009) and Chinese (Mayes et al., 2010) faces. Digitally reducing skin wrinkles, skin sagging, or skin color heterogeneity was shown to improve youthful appearance in Chinese women (Porcheron et al., 2014) and in Caucasian women (Fink et al., 2006; Fink and Matts, 2008; Samson et al., 2010). Other facial criteria have been shown to contribute to age perception but the studies were conducted only with Caucasian faces and Caucasian observers: skin color (Nkengne et al., 2008), facial shape (Lai et al., 2013), internal features (George and Hole, 1998), sclera color (Russell et al., 2014) and hairstyle and color (Rexbye and Povlsen, 2007; Gunn et al., 2009). Studies showing experimentally the cross-cultural validity of facial cues to a youthful appearance are lacking, as is work with racial groups other than Caucasians and East Asians.

The size and shape of internal features of the face are relevant to the perception of age (George and Hole, 1998; Nkengne et al., 2008; Gunn et al., 2009). The color of the lips, eyes and eyebrows was shown to vary with age in a few studies (Wee et al., 2013; Russell et al., 2014), and variations of facial skin color with time have been largely described. The difference in luminance and color between the eyes, mouth and eyebrows and the skin surrounding these features, named facial contrast, has been recently added—to the list of known signs of aging (Porcheron et al., 2013). Facial contrast had been previously shown to be sexually dimorphic in Caucasian and East Asian faces, and to be a cue for perceiving sex from the face (Russell, 2009; Jones et al., 2015). In Porcheron et al. (2013), some aspects of facial contrast were observed to vary with age in Caucasian female faces and were also significantly correlated with the perceived age of the faces. It included the luminance contrast around the eyes and eyebrows, the red-green contrast around the mouth and eyes, and the yellow-blue contrast around the eyes. In addition, individual faces of Caucasian women were perceived as younger when these aspects of facial contrast were artificially increased, but older when these aspects of facial contrast were artificially decreased. Therefore, we have suggested that by increasing facial contrast, cosmetics increase facial femininity and youthfulness of female faces, with a larger effect in older faces.

Here we tested the hypothesis that facial contrast is a cross-culturally valid cue for age perception. This general question contains two parts: does the decrease of facial contrast observed with age generalize to women of different races or ethnicities, and do people from different cultures use facial contrast as a cue for perceiving the age of the face?

**Figure 1:**
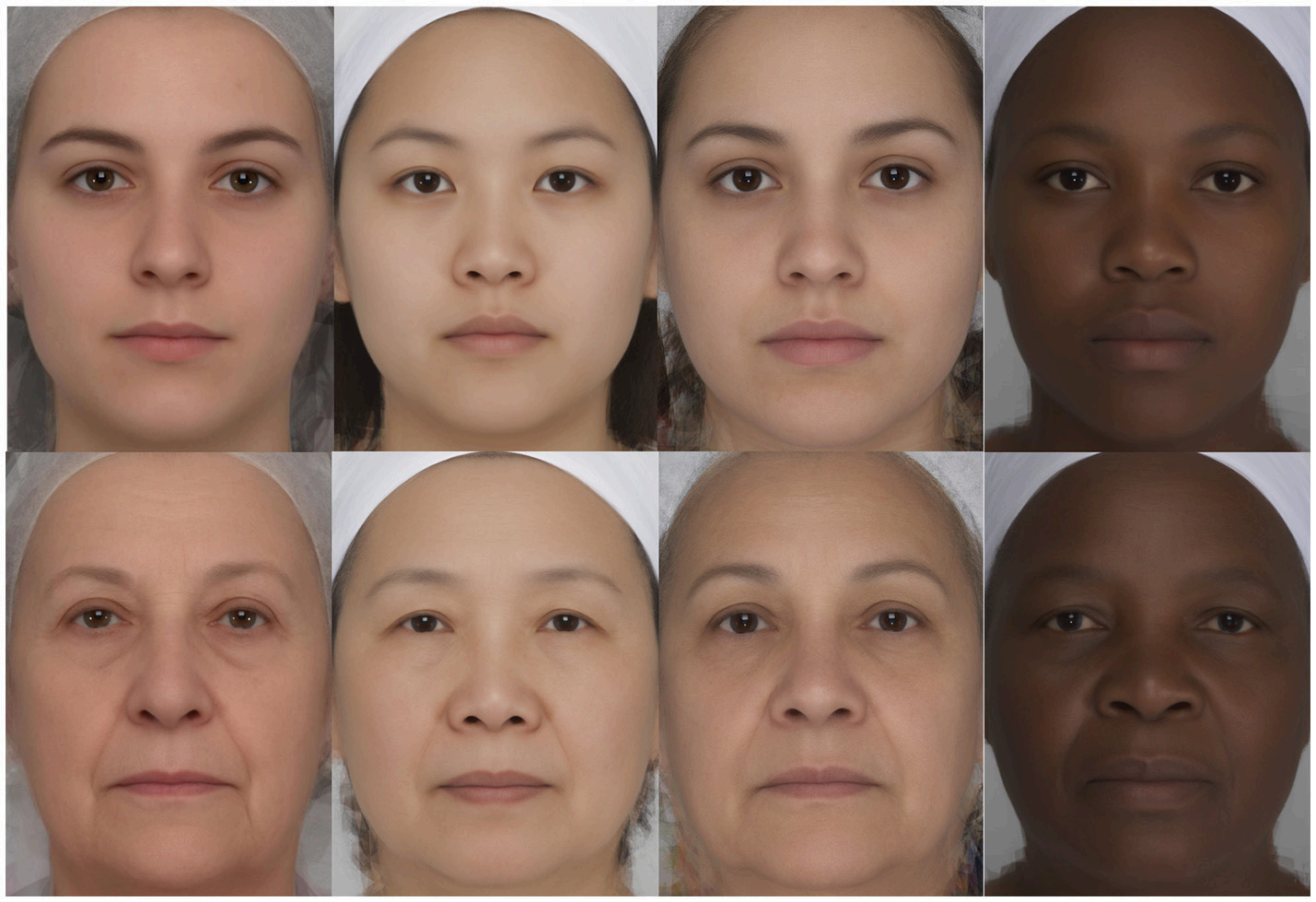
Average faces of young (20–29) and old (60–69) women from French Caucasian, Chinese, Latin American, and South African origin. Each average face was created with 20 individual faces.

Despite the variation in skin color between races (Jablonski and Chaplin, 2000), the changes in skin color observed with age appear to be quite similar in all races. These changes with age are due to the effects of the intrinsic mechanisms of aging and the long term-effects of UV on unprotected areas (chronic sun exposure). The main changes in facial skin color with age are related to chronic sun exposure which causes an overall darkening of the skin in all racial skin types; although some minor modifications of the skin color with age have been reported to be specific to one race or another. For example, photoaging leads to facial skin yellowing in Asians and facial skin reddening in Caucasians (Cazorla, 2014). The effect of the intrinsic and extrinsic factors on facial features across races has been little studied. Therefore, it was difficult to predict how facial contrast varies with age across races solely on the basis of the literature. We generated composite faces by age and by race in order to define a prediction. From the average faces in Figure 1, it appears that there is a global decrease of facial contrast with age in women from different races. The four older composites seem to have lower contrast than the four corresponding younger composites. Nevertheless, the way that aspects of facial contrast decrease with age may vary slightly by race.

Therefore, our first hypothesis is that facial contrast of all regions of the face should decrease similarly across faces of different races or ethnicities. If there is a global decrease of facial contrast with age in female faces of different races or ethnicities, we expect facial contrast to be a relevant cue for age perception independent of the ethnic origin of the face and the cultural origin of the observer.

In the first study, we measured facial contrast using sets of Chinese, Latin American and black South African female faces to determine whether facial contrast decreases with age in these three ethnic groups of faces, similarly to the Caucasian faces measured in Porcheron et al. (2013). We used only female faces in order to hold constant the gender variable that has been shown to predict facial contrast in different racial groups (Russell, 2009; Jones et al., 2015). Indeed, we sought to isolate the relationship between facial contrast and age according to ethnicities. In the second study we tested experimentally whether facial contrast is causally related to perceived age independent of the ethnic origin of the face and the cultural origin of the observer. We manipulated the aspects of facial contrast that were found to vary similarly in the four groups of faces in order to create new versions of the faces with either increased or decreased facial contrast. In a forced choice test design, French and Chinese participants were shown both images and asked to select which face looked younger.

## Study 1: does facial contrast change with age in different ethnic groups?

### Materials and methods

#### Set of faces

Full face color images of 763 women from diverse ethnicities and with healthy skin were used: 139 Chinese Asian women living in Paris, France (20–78 years old; 41.6 ± 13.8 years old), 180 Latin American women living on the East Coast of the United States (a racially heterogeneous group, 20–80 years old; 44.8 ± 16.2 years old), 155 black South African women living in Pretoria, South Africa (20–70 years old; 45.8 ± 15.0 years old) and 289 French Caucasian women living in Paris (20–70 years old; 45.66 ± 13.2 years old) that were analyzed in Porcheron et al. (2013). Among the Chinese women, 67% were born in China (48.1 ± 11.7 years old) and have spent 21.9 ± 11.9 years in France, and 33% were born in France (21.9 ± 6.9 years old). The Latin American group was composed of women from different countries of origin; 81% of them were born in Brazil, Columbia, Mexico, Puerto Rico, Dominican Republic or the USA, and 19% in other countries of South America or Central America.

These images were acquired using a closed photographic system that allows accurate and reproducible positioning of the subjects as well as controlled lighting conditions. See Porcheron et al. (2013) for the details of the photographic system. The subjects wore no make-up or adornments. The vast majority of the subjects (737 out of 763 totals) wore a headband to keep their hair away from their face. Subjects' eyes were open, and they were asked to keep a neutral expression and gaze directly into the camera. Faces wearing permanent make-up or colored contact lenses were not included. The images were cropped to leave the face contour visible.

Because this was a cross-sectional study, we wanted to determine whether any changes of eye contrast with age could be due to differences in iris color between the young and the older women of our study. This applied only to French Caucasian and Latin American women who presented iris color ranging from blue to dark brown. Toward this end, the iris color of each face of these 2 groups was evaluated using the system described by Seddon et al. (1990). We analyzed the difference in eye color between the older and younger faces in each set of images with a χ^2^ test, and found no significant difference in iris color between the different 10-year age classes in both samples of faces (*p* = 0.65 for Caucasian and *p* = 0.43 for Latin American).

#### Facial contrast measurements

**Figure 2:**
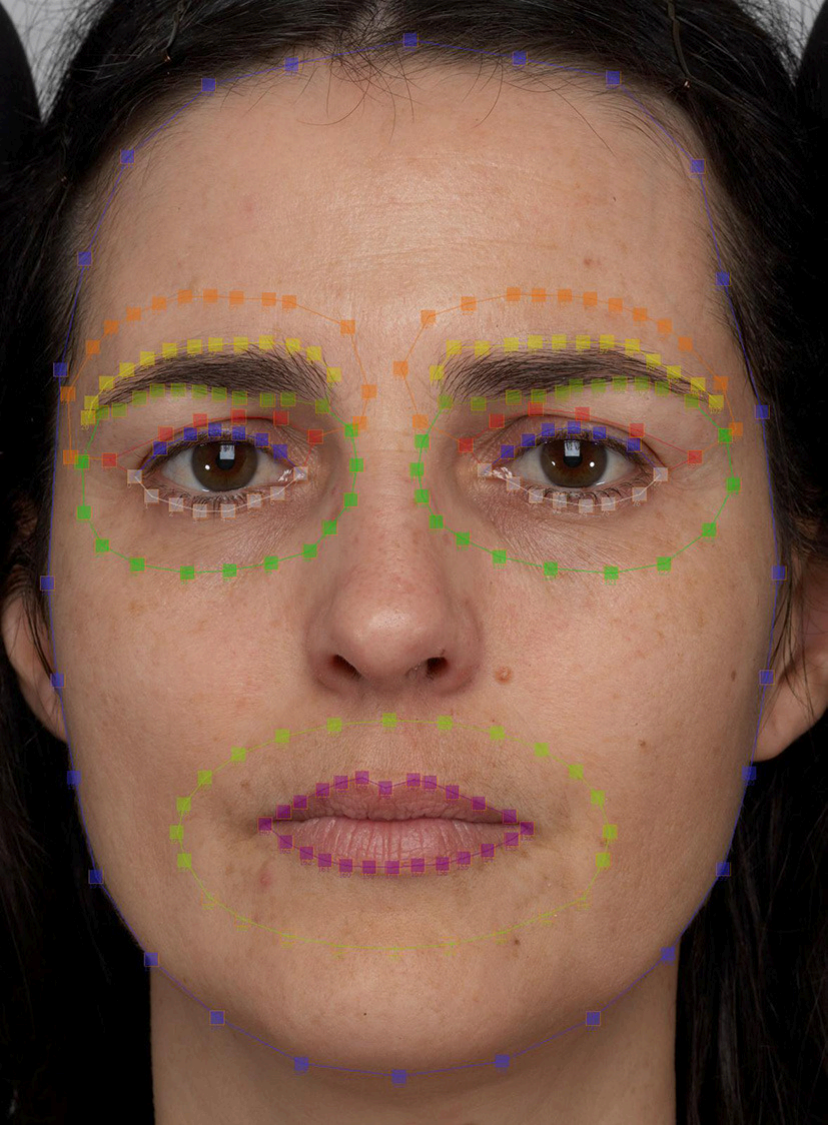
Labeling of facial regions. The dashed lines demonstrate how the features and surrounding skin were defined (eye regions: blue and white dots, skin around the eyes: green dots, mouth region: purple dots, skin around the mouth: light green dots, eyebrow regions: yellow and light green dots, skin around eyebrows: orange and red dots). The individual whose face appears here gave consent for her likeness to be published in this article.

Facial regions were labeled similarly to Porcheron et al. (2013) and corresponded to the eyes, the lips, the eyebrows, annuli surrounding the eyes, an annulus surrounding the lips and annuli surrounding the eye brows (Figure 2). After labeling, we measured the contrast between the eyes and the surrounding skin, the eyebrows and the surrounding skin, and the mouth and the surrounding skin, in the CIELab L^*^ (luminance), a^*^ (green–red), and b^*^ (blue–yellow) axes, using exactly the same method as described in Porcheron et al. (2013) and Jones et al. (2015). The measurement of the contrast was performed using MATLAB 7.8.0 (R2010a). Luminance values of all pixels within the eyes were averaged, as were all the pixels in each of the other features and the annuli surrounding each feature. In this way, a single mean pixel value for each face region (whether a feature or the surrounding skin) was measured for each of the three color channels. The contrast was calculated for each feature as C_f_ = (skin luminance − feature luminance)/(skin luminance + feature luminance). This is an adapted version of Michelson contrast, which varies from −1 to 1, with higher absolute values indicating greater contrast, and 0 indicating no contrast. The same method was applied to measure red-green and yellow-blue facial contrast, with a^*^ ranging from 0 (green) to 255 (red) and b^*^ ranging from 0 (blue) to 255 (red).

### Results

The relationship between age and each aspect of facial contrast according to ethnic origin of face was tested using a Pearson correlation. Results are presented in details in Table 1. A negative coefficient of correlation indicates that the aspect of facial contrast decreases with age, whereas a positive coefficient indicates an increase of contrast with age.

**Table 1:** The relationship between age and luminance (L^*^) or color (a^*^, b^*^) contrast for the 4 groups of faces.

**Feature**	**Contrast**	**Caucasian**			**Chinese**			**Latin American**			**South African**		
		***R***	***P*-value**	**Direction**	***R***	***P*-value**	**Direction**	***R***	***P*-value**	**Direction**	***R***	***P*-value**	**Direction**
Brow	L^*^	−0.50	<0.05	Decrease	−0.33	<0.05	Decrease	−0.49	<0.05	Decrease	−0.21	<0.05	Decrease
	a^*^	−0.27	<0.05	Decrease	0.21	<0.05	/	−0.06	ns	/	−0.36	<0.05	Decrease
	b^*^	−0.23	<0.05	Decrease	0.08	ns	/	−0.11	ns	/	−0.26	<0.05	Decrease
Eyes	L^*^	−0.11	<0.10	Decrease	−0.09	ns	/	−0.13	<0.10	Decrease	−0.25	<0.05	Decrease
	a^*^	−0.32	<0.05	Decrease	−0.10	ns	/	−0.30	<0.05	Decrease	−0.46	<0.05	Decrease
	b^*^	−0.24	<0.05	Decrease	−0.18	<0.05	Decrease	−0.17	<0.05	Decrease	−0.40	<0.05	Decrease
Mouth	L^*^	0.03	NS	/	0.21	<0.05	Increase	0.14	<0.10	Increase	0.18	<0.05	Increase^a^
	a^*^	−0.292	<0.05	Decrease	−0.35^b^	<0.05	Decrease	−0.34^b^	<0.05	Decrease	0.23^c^	<0.05	Decrease
	b^*^	0.16	<0.05	Increase	0.28	<0.05	Increase	0.06	ns	/	−0.06	ns	/

a The L^*^ mouth contrast of South African faces decreased until around 40 years old but then increased with age.

b The a^*^ mouth contrast was analyzed in absolute value for the Caucasian, Chinese and Latin American faces, because it had negative values for all these faces.

c The a^*^ mouth contrast of South African faces had positive and negative values according to the face; the slop of the correlational line increased when the age increased but in a negative value range (so closer to 0 in older faces than in younger faces). The aspects of contrast that decreased with age in the 4 groups of faces are highlighted in dark gray, and the aspects of contrast that decreased with age in 3 of the 4 groups of faces are highlighted in light gray.

Several aspects of facial contrast were found to significantly decrease with age in all four samples of faces. Specifically, the luminance contrast around the eyebrows, the a^*^ (green to red) contrast around the mouth and the b^*^ (blue to yellow) contrast around the eyes significantly decreased with age in all faces. The luminance contrast around the eyes and the a^*^ contrast around the eyes decreased with age in all faces, although it was not significant in Chinese faces.

There were also a few changes in contrast that were specific to one group of faces or another. For instance, the luminance contrast around the mouth increased with age in the Chinese faces and tended to increase with age in Latin American faces, though it did not change in the black South African or French Caucasian faces. The yellowness contrast around the mouth increased with age in Caucasian and Chinese faces but did not change in the Latin American and black South African faces.

In summary, most aspects of facial contrast were found to decrease with age in the four sets of faces; three groups that were ethnically homogeneous and one group which was ethnically diverse (Latin American faces). The decrease with age appeared to be stronger in Caucasian and South African faces than in Chinese and Latin American sets which were more diverse in the relationships between facial contrast and age.

## Study 2: is facial contrast a cross-culturally valid cue to age perception?

Having found that several aspects of facial contrast decrease with age in all groups of faces we aimed to determine whether facial contrast influences the perception of age independently of the ethnic origin of the face and the cultural origin of the observer. We tested 4 ethnic origins of faces (the same origins as in study 1) and 2 cultural origins of participants (French Caucasian and Chinese Asian). We manipulated the aspects of facial contrast that decreased with age across ethnicities, to test whether they are causally related to age perception. We considered any of the contrast which has the same change with age in at least 3 groups of faces in order to look at what was consistent in most of the groups, namely the luminance contrast around the eyes and eyebrows, the a^*^ contrast around the eyes and mouth, and the b^*^ contrast around the eyes. These five aspects of facial contrast were either increased or decreased to create 2 versions of each face, a high and a low contrast version. Then we used a forced-choice test design in which the two versions of each face were compared side by side by the participants.

### Materials and methods

#### Stimuli

Ninety-two images were randomly selected from the set of 763 used in the first study in order to have 23 women per ethnic group. For each ethnic group, the 23 faces were homogeneously distributed by age as described in Table 2.

**Table 2:** Age distribution of Study 2 faces by ethnic origin.

**Age**	**Caucasian**	**Chinese**	**Latin American**	**South African**
N	23	23	23	23
Mean ± SD	43.00 ± 12.92	42.35 ± 12.82	41.83 ± 13.37	42.87 ± 14.02
Min-Max	23.0–63.0	20.0–63.0	20.0–66.0	21.0–67.0

Each face was manipulated to increase or decrease the aspects of facial contrast that were observed to decrease with age or decrease with the age in the majority of the four ethnic groups. Specifically, we manipulated the luminance contrast around the eyes and eyebrows, the a^*^ contrast around the eyes and mouth, and the b^*^ contrast around the eyes. To manipulate contrast around a feature, the features were manipulated while the surrounding skin was left unchanged. The direction and the magnitude of the manipulations are summarized in the Table 3.

**Table 3:** Direction of the manipulations for each version (low contrast vs. high contrast) and magnitude of the manipulations by ethnic origin of faces.

**Feature**	**Color channel**	**Direction of the manipulation**		**Magnitude of the manipulation (Mean** ± **SD)**			
		**Low contrast version**	**High contrast version**	**Caucasian**	**Chinese**	**Latin American**	**South African**
Brows	L^*^	Lighter	Darker	17.84 ± 3.05	19.21 ± 4.34	17.02 ± 3.69	11.09 ± 3.16
Eyes	L^*^	Lighter	Darker	11.22 ± 2.99	10.13 ± 1.74	10.69 ± 2.25	7.79 ± 2.06
Eyes	a^*^	Redder	Less red	2.32 ± 0.98	2.41 ± 0.62	2.99 ± 0.64	2.23 ± 0.49
Eyes	b^*^	Yellower	Less yellow	4.75 ± 0.69	4.49 ± 0.72	4.90 ± 0.63	4.99 ± 0.99
Mouth	a^*^	Less red	Redder	4.86 ± 1.31	3.74 ± 0.53	3.98 ± 0.82	3.08 ± 0.71

The magnitude of the manipulations corresponds to the mean absolute difference in a^*^, b^*^ or L^*^ of the feature between the high and the low contrast faces.

**Figure 3:**
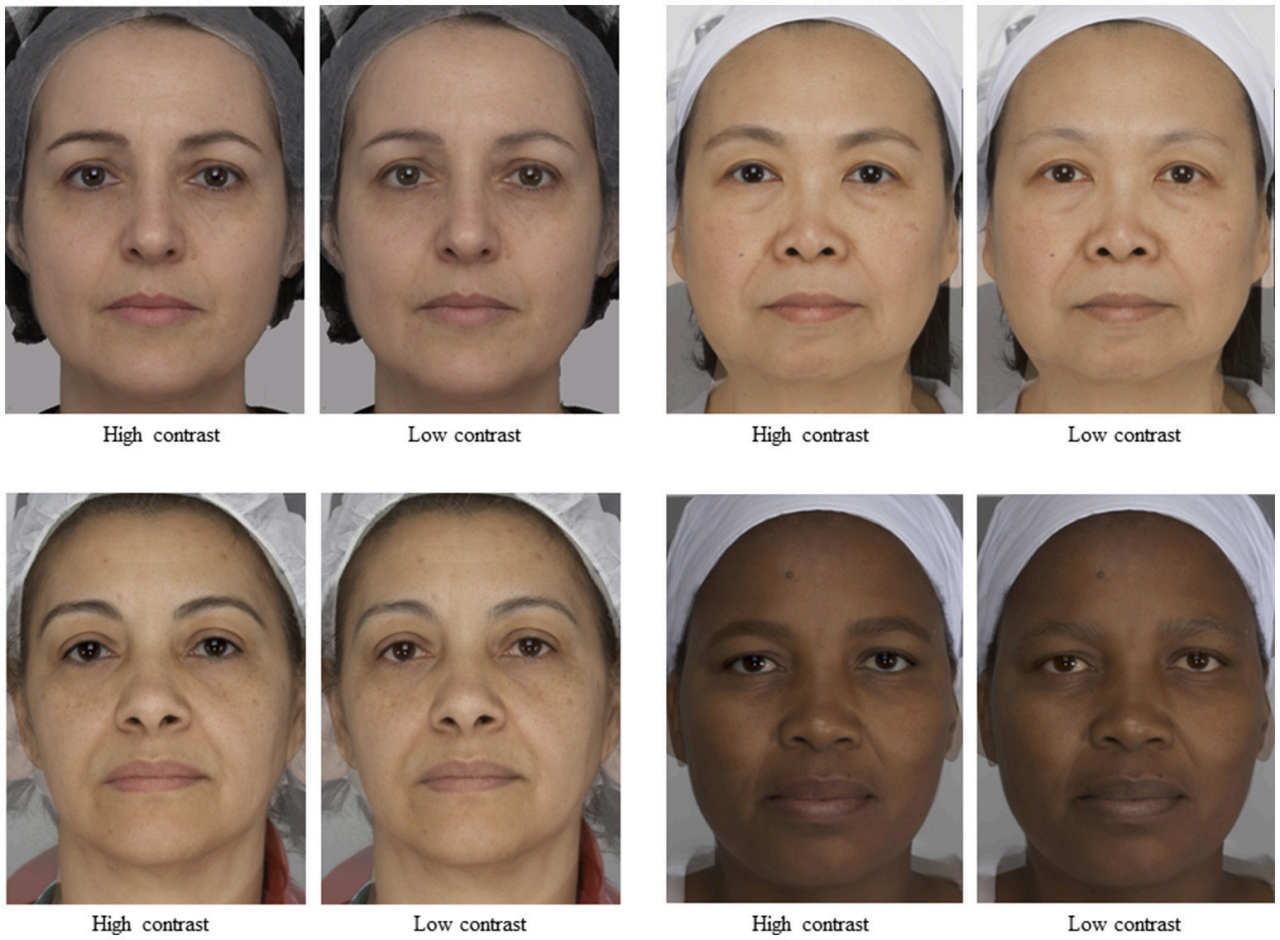
Examples of high and low contrast versions for Caucasian, Chinese, Latin American and South African faces. To respect the privacy of the women, we have averaged individual faces from the study to create 4 composites in high vs. low version.

The procedure of manipulation was similar to the one used in Porcheron et al. (2013). The burn tool in Adobe Photoshop® was used to selectively darken the eyebrows and the dodge tool was used to selectively lighten the eyebrows. To manipulate all other feature contrasts (i.e., the a^*^, and b^*^ contrast between the eyes and the surrounding skin and the a^*^ contrast between the mouth and the surrounding skin) we individually manipulated the L^*^, a^*^ or b^*^ channel (0–255) of the relevant feature without changing the rest of the face. For each feature and each face, we selected the magnitude of change that seemed to maximize the change to apparent age while maintaining a naturalistic appearance. The magnitudes of manipulation were weakened for some features of some faces in order to maintain a naturalistic appearance. The critical point is that the direction of change was consistent across all faces. Example stimuli are shown in Figure 3.

#### Participants

The 92 pairs of faces were shown to 2 groups of participants. The first group was composed of 108 French Caucasian participants who did the test in Paris (59 females: 20–59 years, 38.8 ± 11.4 years; 49 males: 21–64 years, 41.5 ± 12.2 years). The second group was composed of 74 Chinese participants who did the test in Hangzhou (40 females: 18–70 years, 42.8 ± 17.9 years; 34 males: 17–74 years, 44.6 ± 19.2 years).

#### Forced-choice test design

For each of the 92 stimuli faces, participants saw both the high contrast and the low contrast versions presented side-by-side and indicated with a button press which version looked younger. The presentation of the faces was randomized and counterbalanced by age group, manipulation [low/high] and ethnic origins. The pairs of faces were presented without limit of time. Two different monitors were used for the French study, they were both calibrated. The Chinese participants were all presented the stimuli on the same monitor, this monitor was not calibrated. In both locations, controlled lightening conditions were used in the test rooms.

### Results

With regard to the hypothesis that facial contrast is a cross-cultural cue to perceiving age, we did not expect any effect of the ethnic origin of the face and the cultural origin of the participants. However we tested a possible *own-race bias* on the likelihood of choosing the high contrast version of the face. In other words, we tested whether French participants selected the high contrast faces the most often for Caucasian faces and Chinese participants for Chinese faces. According to a recent study, we are more susceptible to perceive a small difference in age between two faces of our own race than between two faces of another race (Anzures et al., 2010). We had no specific prediction about the effect of the age and gender of the participants on the likelihood of choosing the high contrast version of the face.

Because it was a forced-choice task, each dependent variable is binomial (subjects selected either the high or low contrast version of the face), and so we performed a factorial logistic regression model with repeated measurements (faces and participants) to investigate the effect of ethnic origin of face and cultural origin of participant as effects on the likelihood of choosing the high contrast version of the face. See Russell et al. (2014) for more details on the statistical method.

**Figure 4:**
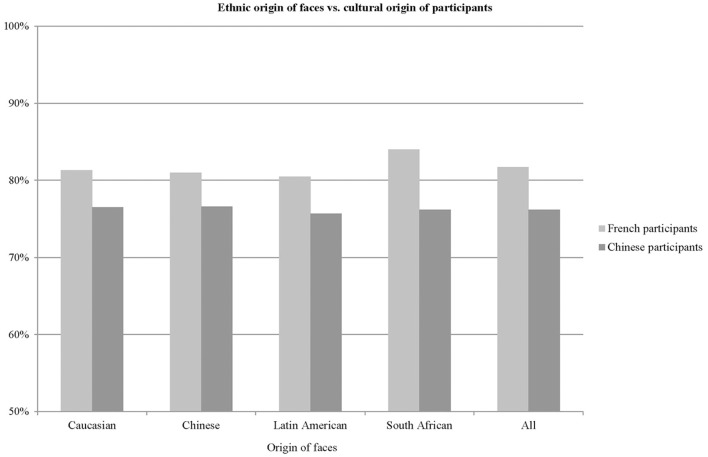
Percent of trials for which the high contrast face was judged younger than the low contrast face. Chance performance is 50%.

Overall, participants selected the high contrast face in 79.2% of the trials. These results are shown in Figure 4, as a function of the ethnic origin of the faces and the cultural origin of the participants. The high percentages observed for French and Chinese participants for each group of faces (Caucasian faces: 80.2 vs. 77.4%, Chinese faces: 79.1 vs. 77.6%, Latin American faces: 80.4 vs. 76.5%, South African faces: 82.3 vs. 77.2% for French and Chinese participants respectively) indicates that faces with increased facial contrast were perceived as younger, regardless of the cultural origin of the participants and the ethnic origin of the faces (own-race and other-race faces). There was a significant main effect of the cultural origin of the participants (χ^2^ = 26.73, *p* < 0.0001). The effect of the manipulations was significantly but slightly stronger in French participants than in Chinese participants, regardless of the ethnic origin of the face. There was no effect of the ethnic origin of the face and no interaction effect as predicted.

## General discussion

These studies addressed the general question of whether facial contrast is a cross-culturally valid cue for perceiving age from the face. This general question contains two parts: does facial contrast decrease with age in women of diverse races or ethnicities, and do people from different cultures use facial contrast as a cue for perceiving the age of the face? Using four different samples of faces drawn from four different racial and ethnic groups on four different continents, we tested the hypothesis that aspects of facial contrast decrease with age. Then with two different participant groups drawn from different racial groups and different cultures on two different continents, we tested the hypothesis that people use facial contrast as a cue for perceiving age from the face. We found support for both hypotheses with all our face and participant samples. First, there were consistent decreases in facial contrast in all four face samples that we tested. Second, both participant groups perceived faces with increased facial contrast as younger than the same faces with decreased facial contrast. These results are both consistent with the idea that facial contrast is a cross-cultural cue for perceiving facial age. While we confirmed that aspects of facial contrast decrease with age in Caucasian, Chinese, Latin American and black South African female faces, there was also some variation. Three of the nine aspects of facial contrast decreased with age in all four groups: the luminance contrast around the eyebrows, the red-green contrast around the mouth and the yellow-blue contrast around the eyes. Two aspects of facial contrast were decreased or tended to decrease with age in the majority of faces but not in the Chinese set. These included the luminance contrast around the eyes and the red-green contrast around the eyes. Finally, four aspects of facial contrast varied with age in diverse ways according to the ethnic origin of the face. Those were the luminance and yellow-blue contrast around the mouth and the red-green and yellow-green contrast around the eyebrows. It is also noteworthy that most aspects of facial contrast (seven of nine) decreased with age in the Caucasian and black South African faces, whereas the changes observed with age were more diverse in the Chinese and Latin American faces.

The results from Study 2 gave clear evidence that aspects of facial contrast are used as cues for perceiving the age of female faces in both French and Chinese participants. When we tested these groups with faces from the four racial groups and from a large range of age, female faces with increased facial contrast were judged younger than those with decreased facial contrast. The effect was robust and largely above chance regardless of the ethnic origin of the face or the cultural origin of the participant; with French participants selecting high contrast faces slightly more often than Chinese participants. Collectively, these findings are consistent with the idea that the aspects of facial contrast that decrease with age in the majority of female faces are cross-culturally valid cues to the perception of women's age.

While aspects of facial contrast decrease with age in all women, the application of cosmetics serves to increase the luminance and color portion of facial contrast (Russell, 2009; Etcoff et al., 2011; Jones et al., 2015). For example, the application of lipstick increases red contrast around the mouth (Jones et al., 2015), which was found here to decrease with age in all four racial groups. The present work advances our knowledge of how cosmetics are applied by women to enhance their beauty, by increasing the contrast between the facial features and the surrounding skin. This visual feature is related to two known factors of beauty: age and sexual dimorphism. By increasing this feature, cosmetics can alter an important universal factor of beauty and make women look more feminine and younger. This provides evidence for biological influences on the pattern of cosmetic use, in addition to cultural influences.

The scientific study of the ways that makeup changes the appearance of the face has begun only recently, and there is little published research on the topic. To our knowledge there are no studies that have systematically compared the ways in which women of different racial groups use makeup. We predict that women of a particular race use makeup to modify (or *should* use makeup to modify) their facial contrast in the ways that facial contrasts change with age in their particular race. Here we observed some specificity according to races in the way aspects of facial contrast vary with age (i.e., luminance and yellow contrast around the mouth). Only Chinese women and Latin American women have higher luminance contrast around the mouth with age; their lips appear darker with age. These observations suggest possible reasons for cultural preferences in makeup shades used by women and that the shades of lipstick may have different effects on perceived age in women from different races and cultures. Because we observed some similarities but also some specificities in the way facial contrast varies with age according to the origin of women; this points toward a rational approach to the development of cosmetics and to the personalization of cosmetics.

Although the evidence from this work is clearly consistent with cross-culturality, the findings will need to be extended to other groups of *participants* (e.g., South Africans, Latin Americans) and *target faces* of other races and ethnicities. Finally, our focus here was the difference between races rather than gender. Because of the known sex differences in facial contrast, we sought to test large samples of faces that differed in terms of race but not gender. A recent study showed that the decline of facial contrast is very similar in male and in female faces (*Caucasian population*) (Russell et al., 2017). Future work will have to confirm whether facial contrast is also a cross-cultural cue for perceiving the age of male faces. Similarly, we have no evidence regarding changes in facial contrast before the age of 20 or after the age of 70, or 80 depending on the sample of faces. All of these questions deserve to be investigated in order to provide evidence for the universality of facial contrast cues to age.

## Conclusion

This is the first study in the age perception research field in which large samples of faces from diverse races taken around the world are tested with participants of different racial and cultural origins. We have shown that several aspects of facial contrast decrease with age in all racial groups, though some other aspects of facial contrast vary with age in racially-specific ways. Globally, older faces have less facial contrast than younger faces. We have also found that artificially increasing those aspects of facial contrast that decrease with age in diverse races and ethnicities makes the faces look younger, independent of the ethnic origin of the face and the cultural origin of the observers. These findings are consistent with the idea that facial contrast is a cross-culturally valid cue for perceiving age.

## Ethics statement

All study participants have granted their written informed consent in accordance with Helsinski declaration. In France, this study required the information of the National Data Protection Authority or CNIL which is in charge of ensuring respect for the French law on data processing, data files and individual liberties. The consent of the study models and participants was required before processing personal data. Informed consent ensures both respects for voluntary participation in the study and for the person's right to privacy and data protection consistent with the requirements of applicable law French national data protection authority CNIL. In addition, all models who participated in this study signed an informed consent form, stating that their facial images could be used by CE.R.I.E.S for research purposes or used for research under the CE.R.I.E.S responsibility. CE.R.I.E.S is the skin research center of Chanel PB. Study 2 was approved by an ethical comity in Zhejiang Sci-Tech University.

## Author contributions

Conception and design of the research: AP, RR, and FM. Acquisition of the data: AP, YL, and LG. Data analysis: EM and FS. Interpretation of the results: AP, YL, LG, OP, RR, and FM. Drafting the work: AP. Critical revision of the paper: OP, RR, and FM. Final approval of the version to be published and agree to be accountable for the content of the work: All authors.

### Conflict of interest statement

AP, EM, and FM are employees of Chanel PB, a cosmetic company. The other authors declare that the research was conducted in the absence of any commercial or financial relationships that could be construed as a potential conflict of interest.
